# Supplementary material for: Curated and harmonised transcriptomics datasets of interstitial lung diseases
Source: Data Brief. 2025 Oct 14;63:112139. doi: 10.1016/j.dib.2025.112139 (PMC12581653; doi:10.1016/j.dib.2025.112139)
Supplement: Supplementary file 1 [file mmc1.zip › Supplementary_material/RNA-seq/fastQC reports/GSE184316/SRR15926805_fastqc.html]

SRR15926805.fastq.gz FastQC Report 

FastQC Report

Mon 5 Feb 2024  
SRR15926805.fastq.gz

## Summary

- Basic Statistics
- Per base sequence quality
- Per sequence quality scores
- Per base sequence content
- Per sequence GC content
- Per base N content
- Sequence Length Distribution
- Sequence Duplication Levels
- Overrepresented sequences
- Adapter Content

## Basic Statistics

| Measure | Value |
| --- | --- |
| Filename | SRR15926805.fastq.gz |
| File type | Conventional base calls |
| Encoding | Sanger / Illumina 1.9 |
| Total Sequences | 8169506 |
| Sequences flagged as poor quality | 0 |
| Sequence length | 25-374 |
| %GC | 49 |

## Per base sequence quality

## Per sequence quality scores

## Per base sequence content

## Per sequence GC content

## Per base N content

## Sequence Length Distribution

## Sequence Duplication Levels

## Overrepresented sequences

| Sequence | Count | Percentage | Possible Source |
| --- | --- | --- | --- |
| AGGCTACACACTGAGGACTCTGTTCCTCCCCTTTCCGCCTAGGGGAAAGT | 71788 | 0.8787312231608618 | No Hit |
| CAGTGGGGCCACGAGCTGAGTGCGTCCTGTCACTCCACTCCCATGTCCCT | 71267 | 0.8723538485680774 | No Hit |
| TTACAAAAGCAGAATAAAAGCGAAAAGAAATGAAAATGTTACACTACATT | 67442 | 0.8255333921047369 | No Hit |
| CAATCCACTTGATCCCAACTCATCTCTCATTTATTTCGGCTTCTTTTATT | 63208 | 0.7737065129764272 | No Hit |
| GGGAAGCGGGGAATGTCTACGTGCGTATGCACGTGGCACTCTCTGCCCGA | 56483 | 0.6913881940964363 | No Hit |
| CACCTCCCCGAAGCTCAGGGAGAGCCCTGTTAGGGCCGCCTCTGGCCCTA | 37307 | 0.45666163902688856 | No Hit |
| TGATATGGTTCCTGGCAAGCCCATGTGTGTTGAGAGCTTCTCAGACTATC | 30828 | 0.37735451813120646 | No Hit |
| TGATGACACCCACCGCAACTGTCTGTCTCATATCACGAACAGCAAAGCGA | 30698 | 0.37576323464356354 | No Hit |
| AGACATCAAATCCTGCAGACAAGGGGAGCCCTCAGTCTGCAGGGCTCCAT | 30049 | 0.3678190578475614 | No Hit |
| ATCAAAGAAGAACATGTGATCATCCAGGCCGAGTTCTATCTGAATCCTGA | 24759 | 0.303066060542706 | No Hit |
| CCCTGGCTGCTACAGGAGAATAGCAGACAGGTATAGTTAAGAAACAACTT | 24694 | 0.3022704187988845 | No Hit |
| GGCATCAAAAGTGATGGACTGCCCATTGCTGGAGAAGACCTTCTCTCCTA | 23181 | 0.28375032713116316 | No Hit |
| GGCAATGAGCGGTTCCGCTGCCCTGAGGCACTCTTCCAGCCTTCCTTCCT | 22883 | 0.280102615751797 | No Hit |
| AGCTGCTAAGTGCTGTGTTGTCGTTCCCCCTGCTTAAAATAAAGTTGTTT | 22318 | 0.2731866529016565 | No Hit |
| TACCACTGCCACCTTCTCCATCGGCTCCACTGGCCTCGTGGTGTATGACT | 22203 | 0.2717789790472031 | No Hit |
| GAAACCCAGACACATAGCAATTCAGGAAATTTGACTTTCCATTCTCTGCT | 21445 | 0.26250057225002343 | No Hit |
| CTCTCTTTCTGGCCTGGAGGCTATCCAGCGTACTCCAAAGATTCAGGTTT | 21307 | 0.2608113636246794 | No Hit |
| GGAAGACAGTGTGGCGATTCCTCAGGGATCTAGAACTAGAAATACCATTG | 21129 | 0.2586325293108298 | No Hit |
| GTCCACGTCACACTTCATGATGGAGTTGAAGGTAGTTTCGTGGATGCCAC | 21043 | 0.2575798340805429 | No Hit |
| CTTCATGATGTAGCAGCAGGTGCCAGGGGCTGGCTTGTAGGCGATCAGCA | 20415 | 0.24989271077100625 | No Hit |
| ATTGATTCTTCCTACCCATGAGCATGGAATGTTCTTCCATTTGTTTGTAT | 17751 | 0.2172836399165384 | No Hit |
| GAAGGACCTCTTCAAGGAGAACTACAAACCACTGCTCAAGGAAATAAAAG | 16910 | 0.2069892598157098 | No Hit |
| TTCAGCCCTGATCAAGACATGAGGGAGGCAGGGGCTCAGCTGAAGAAGCT | 16385 | 0.2005629226540748 | No Hit |
| CAGCATGTTCCCTCTCCTCATGAGATTGGTGAAGAAAGTATTTGGCAAAG | 14412 | 0.17641213556853988 | No Hit |
| CAGCAGTTTACACCTGCTAGGGTGTTCAAAGGTCAGTGCTATAGAAATTC | 14255 | 0.1744903547411557 | No Hit |
| ACGTTTACCTGTCCATGTCTTACTACTTTGACCGCGATGATGTGGCTTTG | 13639 | 0.16695011913817065 | No Hit |
| TGCCATATCCACATGGAAAATCTCATCACCATCAAAGTCAAACATAAACT | 13462 | 0.16478352546653371 | No Hit |
| AACAAGCACAAAGCCAAGAAAACCAACGATGCCAGATACTGAATTTCTAT | 13255 | 0.16224971252851764 | No Hit |
| ATAGCAGCATGATTTATAGTCCTTTGGGTATATACCCAGTAATGGGATGG | 12935 | 0.15833270702047345 | No Hit |
| ACAAGAAGTACATCAAAGATTACATGAAATCAATCAAAGGGAAACTTGAA | 12856 | 0.15736569628567504 | No Hit |
| GTTCCATATCAGAGCTGTGATCTTGAGAGCCCTCTCCTTGGCTTTCCTGC | 12370 | 0.15141674417033293 | No Hit |
| TCAGGAGACGCTCGTAGCCCTCGCGCTTCTCCTCGGCCAATTCGCGGAAG | 11127 | 0.1362016259000238 | No Hit |
| CAATGGGGTACTTCAGGGTCAGGATGCCACGCTTGCTCTGGGCCTCGTCG | 10898 | 0.1333985188333297 | No Hit |
| CAATCCACTTGATCCCAACTCATCTCTCATTATTTCGGCTTCTTTTATTC | 10893 | 0.1333373156222665 | No Hit |
| GGCATCAAAAGTGATGGACTGCCCATTGCTAGAGAAGACCTTCTCTCCTA | 10761 | 0.13172155085019827 | No Hit |
| GAAGGACCTCTTCAAGGAGAACTACAAACCACTGCTCAATGAAATAAAAG | 10535 | 0.1289551657101421 | No Hit |
| TCAGGAGACGCTCGTAGCCCTCGCGCTTCTCCTCGGCCAGTTCGCGGAAG | 10460 | 0.12803711754419422 | No Hit |
| CGATCAGCACGGGCTCGTACTCTGCCACAAACTGATCACACTGCTTCTGG | 10153 | 0.12427924038491434 | No Hit |
| TTTTCCATGAGCTTAATGATGCTTTCTCTGGGCTTTTGGGGGAGGGTGTC | 9949 | 0.12178214937353617 | No Hit |
| GGCAATGAGCGGTTCCGGTGTCCGGAGGCGCTGTTCCAGCCTTCCTTCCT | 9499 | 0.11627386037784905 | No Hit |
| GTCCACGTCACACTTCATGATGGAGTTGAAGGTGGTCTCGTGGATGCCGC | 9350 | 0.11445000468816596 | No Hit |
| GTTCTGCAGCCCCTGTCATAAAAGGTTTTACTCTTTCTGGTCTCTGTTCT | 9038 | 0.11063092431782288 | No Hit |
| ACAAACGCGGCATAAGTTGACACATGGTCCGCCTTGATGGCCCCAGCTCC | 8911 | 0.10907636275681784 | No Hit |
| GTCTCAACATCTGTGTTGGGGAGAGTGGAGACAGACTGACGCGAGCAGCC | 8882 | 0.10872138413265135 | No Hit |
| TGTGGATGAATCTTGAACTGAGTTCCACTTGTAAACTTCTTGTTTCTTGT | 8591 | 0.10515935724877366 | No Hit |
| CGTGAATACCAAGACCTGCTCAATGTTAAGATGGCCCTTGACATTGAGAT | 8535 | 0.10447388128486594 | No Hit |
| GCGTCTCTGTCTTCTTCAGTTTCGACTTATCGAATTTCTCGATCTCAGCC | 8309 | 0.10170749614480973 | No Hit |

## Adapter Content

Produced by FastQC (version 0.11.7)
